# Some Thoughts on Sexualities and Research in India

**Published:** 2004

**Authors:** T.S Sathyanarayana Rao

**Affiliations:** * Professor & Head, Department of Psychiatry, JSS Medical College & Hospital, Mysore-570004. tssrao19@yahoo.com


Indian Journal of Psychiatry, 2004, 46(I), 3-4

### Some Thoughts on Sexualities and Research in India

**Dr. T.S. Sathyanarayana Rao** *

*Few topics evoke so much anxiety and pleasure, pain, hope, discussion and silence as the erotic possibilities of our bodies.*

(Crooks and Baur, 1996)

Sexuality is part and parcel of our existence and it matters to a great extent of our living. To quote John Bancroft (1994) 'there are few people for whom sex has not been important…. and many for whom it has played a dominant part in their lives'. It is difficult to define as it encompasses so many aspects of our lives. ' Expression' of our sexuality may be varied. Sexually, though some behaviours are apparent, many behaviours are as subtle as 'the way we walk, talk, dress or deal with life in general'. (Crooks and Baur, 1996). What is emphasised is not only physical aspect of sex but much more important is biological, psychological and social dimension of our existence.

There are many assumptions concerning sexuality based on one's attitudes, which are invariably very conservative or liberal. The subject matter abounds with myths, fallacies, exaggerations, secrecy and value laden judgements. In such a situation, in order to gain accurate knowledge and develop a more complete understanding about sexuality, research is the only answer. Research enables us to test assumptions in a systematic way to support or refute claims. Like other disciplines, researchers who study human sexuality share certain goals as understanding, predicting and controlling or influencing the events that are subject matter of their respective fields. Though first two aspects are easily understandable, there has always been controversies and complications concerning the control of people's behaviour (Crooks and Baur, 1996).

Compared to many other disciplines, the field of sexuality as we understand today is still an 'infant' science, having originated largely within this century. Though we Indians can proudly claim its origin in the works of Vatsayana (Kamasutra) as early as 400 B.C., historically we have the whole range of wisdom in our vedas, puranas and culture (Pande.A & Dane. L, 2000). However, when we look at the modern research methodologies, the pioneering work of Alfred Kinsey who conducted an extensive general survey of American sexual behaviours, took place only in the late 1940's and early 1950's. Even though considerable body of knowledge is accumulating many questions still remain unanswered.

It is well recognized that sexual health is one of the important component of health in general. It is both the cause and effect of varied behavioural and physical disorders. Inspite of this, sexuality research has received only a cursory or negligible interest by Indian researchers. Physicians either show no interest or ignore patients psychosexual concerns (Avasthi & Nehra, 2000). We lack to great extent the reliable measures and the estimates of sexual disorders (Kulhara & Avasthi, 1995), specialized clinics (Rao. TSS, 2000) special groups, (Rao. TSS, 1989), training facilities (Singh et al, 1987) and concerned practitioners (Avasthi et al, 1994).

There are widely prevalent myths, misconceptions and prejudices concerning sex in our culture (Mishra, 1963) and the so-called 'sex specialists' and 'quacks' have added to the self-perpetuating, iatrogenic disorders. The pioneering work in this area was initiated and expanded by Wig (1960) and coined the term 'Dhat syndrome' for a specific culture-bound clinical condition. Our traditional systems of medicine, which still attribute primacy to 'conservation of semen' are held responsible for difficulty in treating these individuals and patients failure toaccept or continue treatment adequately (Singh, 1985, Chadda & Ahuja, 1990). Though many workers have attempted understanding of the above phenomenon and have succeeded in its inclusion as an entity in ICD-10, much needs to be done to delineate 'phenomenology, course and outcome' (Avasthi, 2000). Avasthi, (2000) has reviewed in great detail the work done in our country in the area of guilt associated with masturbation, sexual activity, sexual dysfunction in males and females, sexual aspects of family planning and other sexual disorders. However, compared to the research and literature being generated in the field of sexuality in the western countries, in particular North American and European countries, it is very abysmal. The very large population and varied sexual problems present by themselves should have been a source of intense interest and study. Rao. TSS (2003) made a fervent appeal for "sexualities" as a speciality section of Indian Psychiatric Society to spur

*Professor & Head, Department of Psychiatry, JSS Medical College & Hospital, Mysore-570004. Email: tssrao19@yahoo.com the concern, interest and research in this area. It was emphasized that Psychiatrists and Psychologists with a good training in behaviour and relationship issues, compassion, empathy and the understanding of various nuances of the mind and instincts are better endowed than other specialists of medicine to deal with sexualities to educate the masses, weed out misconceptions for prevention and to treat the problems. This is the only way to keep out the 'quacks' and 'pseudo – specialists' from exploiting the people and patients.

In conclusion, it is clear that the area of sexualities is very vast still to be explored scientifically for proper understanding. There is a need to overcome the indifference prevalent in majority of the specialists and practitioners. It is worthwhile to suggest certain steps that may be of use in the coming days to spur interest in this specialized field.

1. ***A speciality section in Indian Psychiatric Society***, which may bring about the desired sensitivity and involvement of Psychiatric community in the field of sexuality.
2. ***Initiation of research activity*** in varied aspects of sexualities. Many well accepted sexual phenomenon call for elucidation of phenomenology; course, outcome etc. Much of the research available in the Indian context is confined to a great extent to male sexual dysfunction. Female sexuality, other sexual disorders and methods of treatment, which are culture sensitive, are at present highly neglected areas and need attention.
3. ***Strengthening of medical curriculum***. At present, apart from occasional case reviews and some lectures at some centers, hardly any emphasis is made at undergraduate education. Abysmal lack of knowledge and aversion to handle sexual problems by practitioners at district and PHC level can be sourced to this glaring lacuna in our medical curriculum. There is an urgent need to carry out research to include what, when and how much of the topic. The need to introduce sex education at school level itself should be the aim.
4. ***Starting of multi-speciality clinics*** with emphasis on sexuality at all the major medical colleges and tertiary care centers. At present, their existence is too minimal and scattered.
5. ***Strengthening the NGO movement***. The advent of AIDS and other STD's has brought into focus the concern on sexualities. Involving Non Governmental Organizations can be one of the effective ways to reach the masses. Scientific training of lay counselors is rather a necessity.
6. ***Starting of postgraduate centers and courses.*** Specialization has its advantages and can act as nuclei to overcome manpower inadequacy at present to disseminate the knowledge. As the facilities are minimal in our country, the current initiatives on introduction of specialization by the distant and virtual mode may be feasible and worth pursuing.
7. ***Elimination of quackery*** should be high on our agenda. Strengthening the legal framework and mental health laws to achieve the results are needed immediately. Formulating some suitable mechanism to monitor dissemination of scientific information to the public in mass media is needed. Involving administrative and political class to achieve the goal need to be attempted.
8. ***Research*** in establishing the prevalence of various dysfunctions, disorders, comprehensive understanding of the problems and cost effective, community level innovative interventional strategies need to be developed.

### REFERENCES

Avasthi,A., Basu,D., Kulhara,P. and Banerjee, S.T. (1994). Psychosexual dysfunction in Indian male patients : revisited after seven years. Archives of Sexual Behaviour, 23, 685 – 695.

Avasthi, A., Nehra, R., (2000). Mental Health in India 1950 – 2000, Chapter on Sexual Disorders: A review of Indian Research, People's Action for Mental Health , Bangalore

Bankcroft,J., (1994). Human Sexuality and its problems, Churchill Livingstone, Edinburgh.

Chadda, R.K., and Ahuja, N. (1990) Dhat Syndrome: a sex neurosis of the Indian subcontinent. British Journal of Psychiatry, 156, 577-579.

Crooks, R., Baur, K. (1996). Our sexuality, VI edition, Brooks/Cole Publishing Company, Washington.

Kulhara, P., Avasthi, A., (1995). Sexual dysfunction in the Indian subcontinent. International Review of Psychiatry, 7, 231-239

Mishra. R.S (1963) The text book of Yoga Psychology. Julian Press, New York.

Pande, A., Dave, L., (2001). Indian Erotica, Lustre Press/Roli books, New Delhi.

Rao, TSS., (1989)Medical students attitudes to Psychiatry: Interest to specialize in Psychiatry. Indian Journal of psychological Medicine (1989): 12 (2), 23-28.

Rao, TSS., (2000). Approaches to common sexual problems in Medical practice, "Sumana", Mandya

Rao, TSS., (2003). Editorial : Advocating Sexualities as a speciality section of Indian Psychiatric Society, Indian Journal of Psychological Medicine, 26(2), 4

Singh, R. A., Malhotra,S., Avasthi,A. and Pershad, D. (1987). Sexual knowledge and attitude of medical and non-medical students. Indian Journal of Social Psychiatry, 3 (1+ 2), 126 – 136.

Singh, G. (1985) Dhat Syndrome revisited. Indian Journal of Psychiatry, 22, 119-122.

Wig. N.N(1960). Problems of mental health in India. Journal of Clinical and Social Psychiatry, 17, 48-53.

